# Bioprospecting and biotechnological insights into sweet-tasting proteins by microbial hosts―a review

**DOI:** 10.1080/21655979.2022.2061147

**Published:** 2022-04-17

**Authors:** Muhammad Bilal, Liyun Ji, Shuo Xu, Yue Zhang, Hafiz M. N. Iqbal, Hairong Cheng

**Affiliations:** a School of Life Science and Food Engineering, Huaiyin Institute of Technology, Huaian, China; b Joint International Research Laboratory of Metabolic & Developmental Sciences, School of Life Sciences and Biotechnology, Shanghai Jiao Tong University, Shanghai, China; c Tecnologico de Monterrey, School of Engineering and Sciences, Monterrey, Mexico

**Keywords:** Bioengineering, Sweet Proteins, Natural Sweeteners, Thaumatin, Brazzein, Bioproduction, Host Microorganisms

## Abstract

Owing to various undesirable health effects of sugar overconsumption, joint efforts are being made by industrial sectors and regulatory authorities to reduce sugar consumption practices, worldwide. Artificial sweeteners are considered potential substitutes in several products, e.g., sugar alcohols (polyols), high-fructose corn syrup, powdered drink mixes, and other beverages. Nevertheless, their long-standing health effects continue to be debatable. Consequently, growing interest has been shifted in producing non-caloric sweetenersfrom renewable resources to meet consumers’ dietary requirements. Except for the lysozyme protein, various sweet proteins including thaumatin, mabinlin, brazzein, monellin, miraculin, pentadin, and curculin have been identified in tropical plants. Given the high cost and challenging extortion of natural resources, producing these sweet proteins using engineered microbial hosts, such as *Yarrowia lipolytica, Pichia pastoris, Hansenula polymorpha, Candida boidinii, Arxula adeninivorans, Pichia methanolica, Saccharomyces cerevisiae,* and *Kluyveromyces lactis* represents an appealing choice. Engineering techniques can be applied for large-scale biosynthesis of proteins, which can be used in biopharmaceutical, food, diagnostic, and medicine industries. Nevertheless, extensive work needs to be undertaken to address technical challenges in microbial production of sweet-tasting proteins in bulk. This review spotlights historical aspects, physicochemical properties (taste, safety, stability, solubility, and cost), and recombinant biosynthesis of sweet proteins. Moreover, future opportunities for process improvement based on metabolic engineering strategies are also discussed.

## Introduction

Overconsumption of nutritive (caloric) sugars is one of the main dietary problems around the world. A current report reveals that an average American takes approximately 17 teaspoons of added sugar every day, which is almost double the recommended amounts for men (9 teaspoons) and women (6 teaspoons) [1]. This dietary habit is associated with numerous hostile health impacts like high blood pressure, increased risk of obesity, diabetes, and cardiovascular disorders that necessitate global efforts to minimize sugar consumption [2]. The current consumption of sugar accounts for about 11–13% of the total energy intake of adults in Canada [3], which is 17% high in US children and adolescents [4]. Therefore, the US Food and Drug Administration (FDA) has recently (starting 1 January 2020) restructured the Nutrition Facts label compulsion on beverages and packaged foodstuffs to state the quality of sugar inclusion in grams. In the wake of this awareness of sugar intake behavior and the associated health consequences, there is an increasing demand for non-nutritive (low/zero calorie) and safer sugar alternatives. Many sweeteners are available in the market to satisfy the desire of consumers for sweetness, though each sweetener has explicit uses with certain restrictions [5,6].

Artificial sweeteners (ATS) have gained prominence as sugar replacements in several applications; nevertheless, their safety and long-standing health effects continue to be debatable [2]. For instance, the use of ATS alters the host microbiome, reduces satiety, affects glucose homeostasis, and leads to augmented caloric intake and weight increase [7]. Furthermore, a number of health-related effects, like headaches, dizziness, mood changes, and gastrointestinal problems, are also linked with consuming a widely utilized ATS, aspartame [8]. ATS have also been considered an environmental contaminant. Following application in food products, they enter the environmental matrices, and their degradation or transformation may give rise to toxic substances [9]. As a result, a growing interest has been rekindled in producing natural sugar substitutes from natural resources to overcome consumers’ dietary requirements [10].

**Figure 1.**
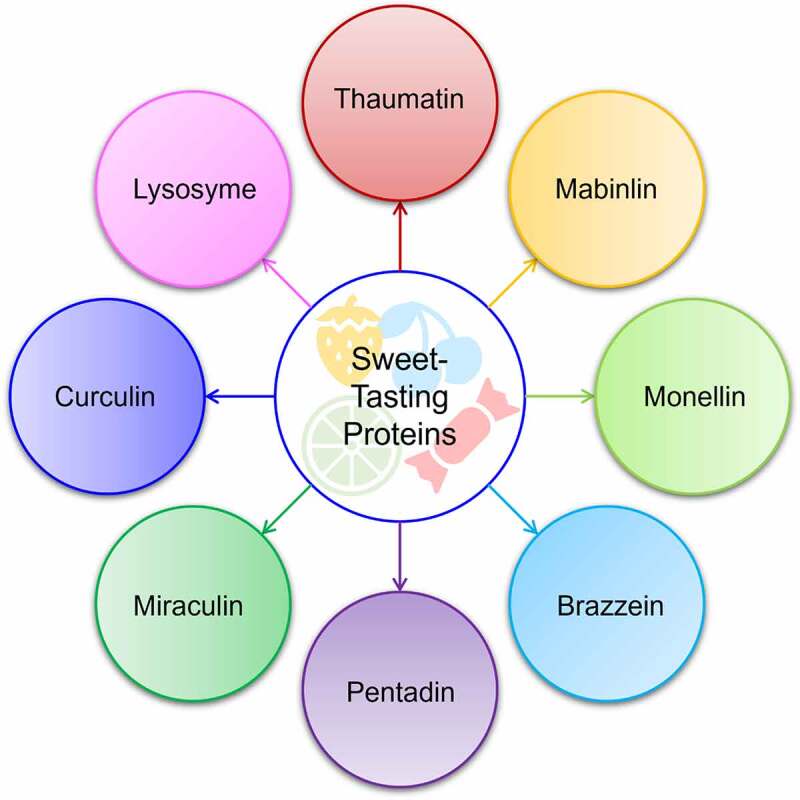
Illustration of sweet-tasting proteins, regardless of their extraction origin, source, and types.

**Figure 2.**
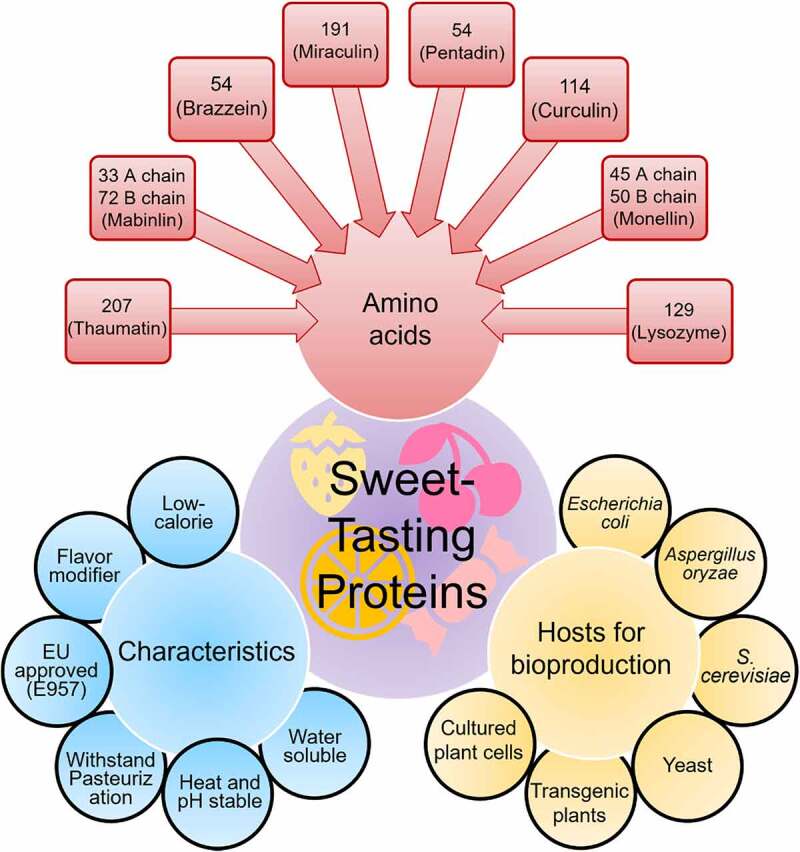
Multifunctional characteristics, amino acids, and bioproduction hosts of sweet-tasting proteins.

So far, various sweet-tasting proteins have been recognized that include thaumatin, mabinlin, monellin, pentadin, brazzein, miraculin, curculin, lysosyme (Figure 1) [21, 34, 43, 64, 67, 94, 101]. All these proteins are originally expressed and isolated in tropical plants except for the lysozyme protein obtained from egg whites. Continuous research for many years on sweetening proteins has explored some of the key characteristics of each protein (Figure 2). Among these proteins, thaumatin has been the most studied, regulated, and marketed sweet protein [11]. Reports have demonstrated that synthetic and natural sweetness are recognized by human T1R2-T1R3 receptors, which are expressed in mouth taste buds, whereas T1R1-T1R3 are involved in recognition of the umami (savory) taste [12]. These receptors possess various binding sites and are triggered by the binding of sweet taste eliciting compounds [13]. Nevertheless, sweet proteins have unique binding features and are unlikely to attach to the same regions, leading to variable sweetness perception [14].

Considering the above critiques, excessive consumption of sugar is one of the main dietary problems in various regions of the world, since this dietary habit is associated with many health complications like high blood pressure, increased risk of obesity, diabetes, and cardiovascular disorders. It is challenging to cut this eating habit; therefore, low-sugar or sugar-free foodstuffs and beverages are highly requisite. The sweeteners making them feasible are high-value-added bio-ingredients. In replacement to sugar (sucrose), the food industry implicates utilizing many intense sweeteners that are mostly synthetic in origin. Customers are keener to consume products composed of naturally derived ingredients with multifunctional health properties without compromising the taste. To accomplish this tendency, food industries have introduced natural sweeteners as alternatives to provide consumers with prospective health benefits. Nature is a prolific source of a plethora of high-value compounds and biomolecules, including sweet-tasting proteins, many of which are yet to be explored. However, it is highly meaningful to execute comprehensive and in-depth scientific investigation to prove the safety of the natural ingredients as food supplements/additives, such as sweeteners

## Natural sweeteners

Food products furnishing a sweet taste typically consist of simple carbohydrates (glucose, fructose, and sucrose) that are metabolized to generate rapid energy sources, as well as complex carbohydrates (i.e., starch) for long-term energy and storage [15]. Nevertheless, the sweet taste can also be brought by glycosides, proteins, D-amino acids, peptides, coumarins, dihydrochalcones, substituted aromatic substances, and other nitrogen-containing compounds [16]. All sweetening compounds bind and trigger a single receptor, TAS1R2-TAS1R3 heterodimer, which comprehends several attachment sites to explicate the variety of compounds inducing sweetness [17]. Currently, sucrose is considered to be the most customarily utilized sweetener, which is available in various refined forms [18]. However, in the past several years, sugar overconsumption has become a problem with grave public health consequences. Reports have shown a clear association of high sugar intake with a higher risk for obesity, type II diabetes, cardiovascular disorders, dental caries, and many other disorders [19]. In this scenario, the inclusion of sweeteners in food products has expanded and become a fascinating option for the scientific and industrial communities.

In contrast to synthetic sweeteners, demand has undoubtedly lied in using natural sweeteners with low-calorie contributions because of increasing consumer’s apprehension regarding the detrimental impacts of a high sugar-containing diet and artificial food supplements. Though numerous low-calorie sweeteners are obtainable, the food industry can use only a few among those due to technological problems and safety concerns [18]. In addition to inducing sweet taste, these compounds are likely to have an influence on product’s flavor, texture, color, and shelf life [20]. Physicochemical attributes, like water solubility, thermal stability, safety, and production cost, are the most critical aspects for selecting a sweetener [21,22]. Its sweetness strength is enormously pertinent, and sweeteners can be categorized based on their origin (natural, synthetic, and semisynthetic) and intrinsic properties (sweetening power, nutritive value) [23]. Sweeteners are also classified into bulk and intense on the basis of their sweetness level compared to an international reference, sucrose (sweetness potency = 1).

Bulk sweeteners possess a likewise or less sweetener potency than sucrose and characteristically impart preservation action, bulk, and texture [24]. They might be applied to breakfast cereals, baked food products, cakes, jams, desserts, preserved foods, sauces, and ice cream [25]. Bulk sweeteners comprise sugar alcohols, like sorbitol, maltitol, lactitol, xylitol, mannitol, isomalt, erythritol, hydrogenated glucose syrups, and hydrogenated starch hydrolyzates [21]. Due to nutritional aspects (e.g., slower assimilation) and functional characteristics (e.g., Maillard reaction), these sweeteners are widely applied to food industries in replacement with sucrose. Intense sweeteners induce a much higher sweetness potency than sucrose [24], and thus a very small amount is needed to attain the desired sweetening level. Such compounds can be natural (e.g., stevioside and rebaudioside), synthetic (e.g., aspartame, sucralose, saccharin, neotame, cyclamate, dulcin, acesulfame-potassiumalitame), or semisynthetic (e.g., neohesperidine dihydrochalcone) [24]. These are widely employed in processed foods, particularly baked food, canned food, sweets, puddings, jellies, and carbonated and non-carbonated drinks [25].

## Sweet-tasting proteins

### Thaumatin

**Figure 3.**
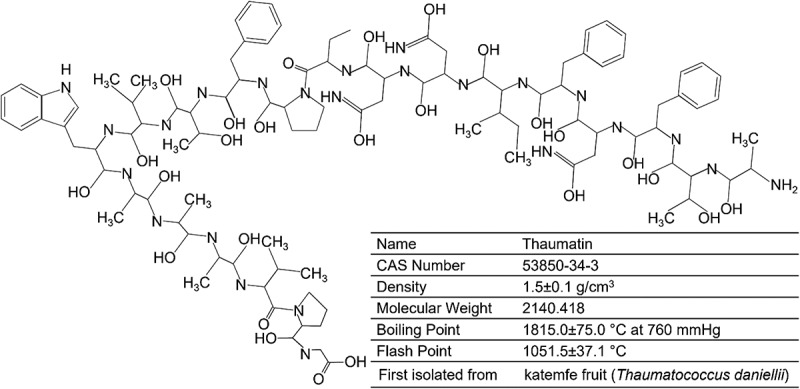
Some important structural and chemical characteristics are thaumatin. *Created with BioRender.com and extracted under premium membership.*

Thaumatins represent a group of very sweet proteins present in the *Thauma-tococcus daniellii Benth* (a tropical plant) fruits. Some important structural,and chemical characteristics are thaumatin are shown in Figure 3. van der Wel and Loeve first isolated the sweet-tasting constituent of *Thaumatococcus daniellii Benth* and acknowledged it as a protein in 1972. Due to their profound sweetness (3000-times that of sucrose, on a weight basis), thaumatins are deemed an attractive sucrose replacement. It is currently isolated from the plant’s fruits and utilized as a sweetening as well as flavor intensifying agent in food and feed products. Thaumatin produces a sweet feeling upon taste by triggering the sweet taste receptors [26]. This protein’s sweet taste is predominantly ascribed to the positive electrical charge distribution on its surface [80,b]. Positive charges at residues Arg76, Arg79 and Arg82, Lys49, Lys67, Lys106, and Lys163, have been reported critical for preserving its sweet taste [27]. After taste stimulus, the thaumatin sweetness can be retained in the oral cavity for about half an hour [28]. Besides, it also shows an objectionable licorice aftertaste because of interactions with bitter taste receptors (T2Rs) [29,30].

Due to challenging extortion from a natural resource, researchers have made attempts to cultivate *Thaumatococcus daniellii Benth* in non-natural habitats. Many efforts have been devoted to producing thaumatin in recombinant microbial platforms as a potential alternative to thaumatin biosynthesis from its naturally occurring source. All thaumatin forms have 207 amino acid residues with a relative molecular weight of 22 kDa and are intensely sweet [28]. The two foremost forms of thaumatin, thaumatins I and II, are different by five amino acids. Recombinant thaumatin has been produced in different microbial strains and transgenic plants by cloning the natural gene for thaumatin II or using an artificial gene containing codons optimized to express in the specific host. Considering the difficulties and high price of thaumatin by natural source extraction, Liu et al. [82] recommended microbial synthesis as the only economically viable if the recombinant microbial strains are capable of producing 1 g of product per liter.

A large number of novel sweetening agents have been introduced in the market, which can serve as a sucrose substitute by displaying the same sensory properties. Before introducing these alternatives in the market, some regulations and directives by the regulatory bodies, like EFSA and FDA, should be followed. The regulatory authorities, such as FDA, SCF, or JECFA of the FAO/WHO, have recommended an adequate everyday intake for those that have been approved for use. Consumption of thaumatin is safe for health unlikely to provoke any tooth deterioration, and thus considered appropriate for diabetic persons compared to other artificial sweeteners [31]. Numerous studies related to safety demonstrate that thaumatin does not induce toxicity or allergenicity.

Hahm and Batt [39] corroborated that thaumatin did not possess any undesirable effects when utilized as a partial sweetener or flavor enhancer within an explicit range. JECFA and SCF further assessed the safety of this sweet protein and granted it as a safe and acceptable ingredient for consumption. It has also been approved as a flavor modifier and an intense sweetener in various countries [32]. FEEDAP (2011) also indicates its safe use for animals and recommends its use as a supplement within a range of 1–5 mg/kg. In addition to being a low-calorie sweetener, it also finds applications in the food industries as a flavor modifier. Prominent uses of this sweetener are additives in animal feeds, dairy, pet foods, and chewing gums [33]. It has also been found to be a suitable sweetener in some other food-stuffs like sweets and ice-creams with an acceptable range of 50 mg/kg. In soft drinks and dairy products, thaumatin is mainly included as a taste modifier [25]. Applying thaumatin is not restricted to imparting sweetness in a wide spectrum of bioproducts, but enhances the flavor and masks unwanted effects in pharmaceuticals and food products [34].

## Bioproduction of thaumatin using different microbial hosts

**Figure 4.**
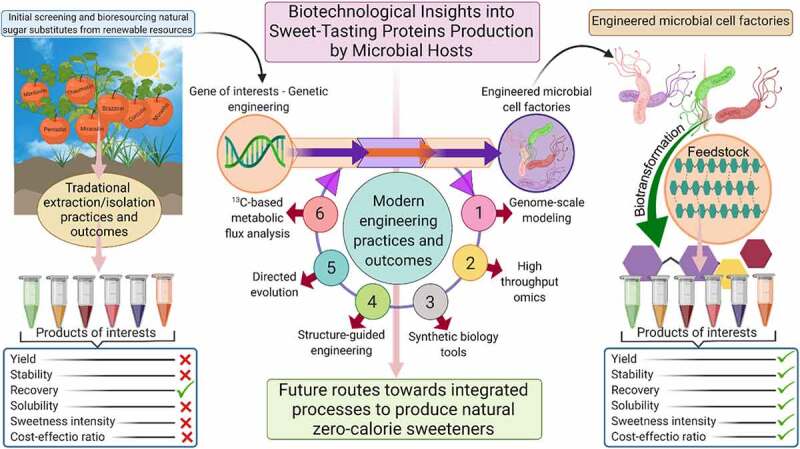
Biotechnological insights into sweet-tasting proteins production by microbial hosts. A comparative overview of traditional extraction/isolation processes and future routes toward integrated processes to extract and produce natural zero-calorie sweeteners with better taste and quality. *Created with BioRender.com and extracted under premium membership.*

Given the growing demand and rising consciousness of the community over non-natural sweeteners, the use of natural sweeteners has garnered increasing recognition. For example, the public favors the inclusion of thaumatin in food products as a prospective replacement for sucrose. Nevertheless, the production of thaumatin from a tropically grown plant hinders its accessibility to overcome escalating demand. In this context, a plethora of studies have been conducted for its production via transgenic plants and genetically engineered microbial hosts for attaining a stable production titer of this protein [44, 75]. Biotechnological insights into sweet-tasting protein production by microbial hosts are shown in Figure 4. Protein and genetic engineering techniques can be applied for large-scale biosynthesis of proteins, which can be used in agricultural, enzyme and biopharmaceutical, food, diagnostic, and medicine industries. Since the production of the first protein (human insulin) by recombinant DNA technology and approval by the FDA in 1982, several recombinant proteins, including albumin, factor VIII, and human growth hormone (HGH) have been introduced in the protein market thanks to the technological advancement for production processes.

The development of *Escherichia coli*, baculovirus, mammalian cell expression, and bioreactor systems has promoted the mass biosynthesis of such proteins. At present, over 400 protein drugs have been attained from recombinant technologies, which are approved and commercialized across the globe. Over 1300 of these proteins are undertaking the approval process [35]. The global market value of recombinant protein was estimated to be US$ 347.2 million in 2016. According to the Coherent Market Insights company (2018), a compound annual growth rate (CAGR) of 6.2% is projected for the period 2017–2025 [36]. Multiple factors should be considered for a low-cost and effective biosynthesis of recombinant proteins. A comprehensive insight into the production pathway and mechanism of thaumatin biosynthesis can improve productivity and yield. A commercially feasible and improved production is required for this protein because of considerable promise as an alternative to the sugar alternatives or less safe sweeteners. Nevertheless, much work is still needed to develop novel methods for synthesizing recombinant proteins on the large scales.

Cloning the target DNA and the protein amplification in the target expression system are two main factors for recombinant proteins expression. Certain properties like the quality, productivity, functionality, and yield of protein are important for assorting the expression system [37]. Many systems are available to express thaumatin, such as yeasts, bacteria, mammals, molds, transgenic plants, and animals. Numerous reports have carried out thaumatin expression in microorganisms. However, the yield was lower, and the resulting bioproduct was not active [38–40]. Saraiva et al. [15] carried out the expression of thaumatin II in *E. coli*, resulting in recombinant protein production as insoluble inclusion bodies. To obtain a soluble, active, and correctly folded protein, renaturation by a reduced/oxidized glutathione system is essential. Varelis et al. [22] used *A. niger var. awamori*-based expression system to secrete thaumatin in a concentration range of 5–7 mgL^−1^. Synthetic genes were introduced into *S. cerevisiae* for thaumatin production using yeast-preferred codons . They applied two different methods to synthesize thaumatin, and obtained a high quantity of sweet-tasting thaumatin [41]. The yield can be increased by using codon optimization and artificial genes. For instance, a satisfactory yield could be attained using artificial genes with optimal codons encoding thaumatin II [42], requiring a renaturation process because the modified proteins were achieved in the form of inactive and insoluble inclusion bodies. As such, the exploitation of manipulated microbial strains is not regarded as a straightforward way, and extensive research is needed to achieve the optimized titer of thaumatin.

## Bacteria

*E. coli* is among the extensively applied host for the expression of the protein [43,44] owing to its rapid expression and growth, easy cultivation, and elevated titers [45]. Moreover, its genetics are well elucidated in comparison with other microorganisms. Notwithstanding appreciable merits, the use of *E. coli* is associated with certain drawbacks, which can probably affect the production efficacy of recombinant proteins. For example, a higher cell density may produce a large amount of acetate harmful to cells. It is also not capable of producing very large proteins. In addition, issues related to difficulties in protein production, disulfide bonds, and refolding ability have also been observed in the *E. coli* system. Failure to synthesize recombinant protein because of the lack of glycosylation is another problem [46]. The modified production in mold, insect, yeast, or mammalian cells presents higher advantages for achieving glycosylation to attain a properly folded and stable protein. Over the years, other bacterial systems are also emerged for producing recombinant proteins, like engineered *Lactococcus lactis* as a good cell factory for expressing membrane protein [47]. They exhibit benefits over *E. coli* in terms of being endotoxin-free and GRAS [48]. *Pseudomonas* species, including *P. aeruginosa, P. putida*, and *P. fluorescens* were also considered preferred substitutes for *E. coli* expression systems to achieve a high titer of recombinant proteins. Laffitte et al. [18] cloned thaumatin II gene into E. coli K12, but the yield of protein was very low. A synthetic gene encoding thaumatin II was effectively expressed in *E. coli* [49]. Saraiva et al. [15] replicated the same system, resulting in about 40 mg of pure thaumatin that showed a comparable sweetness threshold value from the natural source.

## Fungi

A large number of reports are available on production activities using *Escherichia* and *Aspergillus* species. Nonetheless, exploiting yeast and fungal species are preferred because of the complicated *Aspergillus* growth and lower titer in *Escherichia. Pichia pastoris,* and *Saccharomyces cerevisiae* have the capability of producing recombinant proteins with a molecular weight greater than 50 kDa in elevated yields along with glycosylation likelihood. Yeasts can secrete chaperonins, which assist in folding different proteins and handle S–S rich proteins [37]. In contrast to prokaryotic and eukaryotic systems, yeast has more favorable conditions for producing recombinant proteins, leading to higher yields [88]. The compact yeasts genome constitutes a much simpler gene identification process [50]. In addition, yeasts are more robust, faster in growth with a shorter lifecycle (90 min), and amenable to manipulation. They are convenient for fermentation, involving quick growth into higher cell density in simpler media. Furthermore, these eukaryotes are capable of modifying the secreted proteins [89]. Given all these advantageous features, the exploitation of yeast is broadly expanded from the enzyme and chemical preparation to produce an array of valuable biopharmaceutical constituents.

## Yeasts

*Saccharomyces cerevisiae* has been regarded as a prodigious organism for several biotechnological studies. Due to its availability, compatibility, and evident physiological and genetic background, the ethanol industry prefers to use this yeast for fermentative utilization of raw feedstocks like corn, beets, wheat, and sugarcane to produce industrially pertinent bioproducts. It is comparatively easy to genetically manipulate this organism due to the availability of genetic techniques and toolsets. It displays a rapid growth rate in a protein-free medium and is endowed with the ability to make extracellular secretion and post-translational modifications [51,52]. Due to these beneficial attributes, *S. cerevisiae* has been extensively used for heterologous protein expression and other industrial bioprocesses [53]. However, *S. cerevisiae* also presents some drawbacks. For instance, Gellissen and coworkers [88] specified that *S. cerevisiae* tends to hyper-glycosylate heterologous proteins and may lead to batch inconsistencies due to strain instability triggered using episomal vectors. *K. lactis* was also applied to express recombinant thaumatin II but the protein secretion was lower [54]. However, in the recent past, optimized production and quantification of the tryptophan-deficient sweet-tasting protein brazzein in *K lactis* using a chemically defined medium has been reported [55,56]. Using the non-conventional yeasts including *Y. lipolytica, P. pastoris, H. polymorpha, C. boidinii, A. adeninivorans, P. methanolica*, and *K. lactis* might be attractive alternatives to address these inadequacies [57–60]. These yeasts can assimilate methanol as a single carbon source for energy and carbon. Among these, *H. polymorpha* and *P. pastoris* have been widely applied to produce commercially available proteins. Recently, Lee et al. [77] carried out heterologous expression of brazzein in numerous microbial hosts including bacteria, yeast, and transgenic plants. Among all tested systems, it was found that *P. pastoris* appears to be one of the best options for obtaining functional brazzein in high quantities. Like *S. cerevisiae, P. pastoris* also gained in biological and recombinant protein production and is foreseen to have considerable potential for the future. It is a Crabtree-negative yeast that indicates its preference for respiration over fermentation. In contrast to Crabtree-positive yeasts, *P. pastoris* does not assimilate carbon sources to produce ethanol and generates higher biomass, leading to recombinant production of high titer. This fact renders *P. pastoris* a particularly alluring strain for recombinant protein production than *S. cerevisiae*. Recombinant proteins expressed in the *P. pastoris* offer the opportunity for faster and facile production of a large amount of protein that is primarily attributed to easy genetic modifications and robust growth in cheap media leading to higher cell densities. *P. pastoris* has the ability to carry out post-translational amendments, such as glycosylation, protein folding, proteolytic processing and disulfide bond formation [61]. Moreover, the production capability of milligram-to-gram proteins makes it a perfect candidate for the laboratory and industrial production of various recombinant proteins [61–63]. Literature studies have revealed the successful utilization of this strain as a host of choice for producing over 600 recombinant proteins [63,64]. It plays a meaningful role in producing recombinant proteins, particularly complex proteins with disulfide bridges or requiring post-translational modifications [10].

Tomes [70] constructed and expressed a large amount of thaumatin II employing thaumatin gene in the *Pichia* expression system. Thaumatin II gene was ligated with the pPIC9K expression vector containing *S. cerevisiae* prepro α-mating factor secretion signal. Genetic modifications were used to introduce several additional amino acid residues on the C- and N-terminal ends for investigating the contribution of the terminal end region to elicit sweetness in thaumatin molecule. The resultant engineered thaumatin II protein provoked a sweet taste like thaumatin II from the native plant. Co-overexpressing protein disulfide isomerase (PDI) in yeast improved the titer of heterologous proteins expression [65,66]. Global data [35] reported the expression of recombinant thaumatin in *P. pastoris*. A co-expression approach with molecular chaperone results in the generation of engineered variants showing increased thaumatin yield. After thorough purification (42% yield), the recombinant thaumatins were subjected to characterization by size-exclusion chromatography, HPLC, LC-MS/MS for sequence analysis, and tryptophan fluorescence spectroscopies and circular dichroism for conformational characterization. Introducing recombinant C-terminal FLAG-tag and cysteine did not alter the secondary or tertiary structure of thaumatin proteins. Manipulation at these sites might improve the protein’s physicochemical attributes, such as fluorophore attachment and PEGylation for imaging experiments and changed sweet tasting properties, respectively.

## Brazzein

Berlec et al. [72] first isolated brazzein in the obli fruit of *Pentadiplandra brazzeana* Baillon (a west African plant). With a single-chain protein (54 amino acids), brazzein has a relative molecular mass of 6473 with an elucidated three-dimensional structure [67]. It possesses a sweet taste like sugar, which is 500–2000-times sweeter than sucrose [68,69]. The retention of the sweetness profile even after incubating at 353 K for 4 h is ascribed to the compacted structure provided by four disulfide linkages . It has been expressed in transgenic plants [70] and *E. coli* [71]. Studies have reported various transgenic cell lines to synthesize plant-derived brazzein from animal and bacterial cells, such as systems based on *Lactococcus lactis*, yeast, *K. lactis, E. coli*, and mice [72–74].

In earlier reports, recombinant brazzein has been produced and purified via expression in the *E. coli* and *K. lactis*. The recombinant brazzein demonstrated 1800-times greater sweetness compared to sucrose. Its derivatives with mutation of critical residues were even sweeter than the native brazzein. Amongst the mutants, brazzein with three mutations (H31R/E36D/E41A) was 22,500-times sweeter than sucrose and represented 18-fold sweetness than wild-type brazzein [74–77]. Moreover, brazzein produced by the *K. lactis* displayed anti-inflammatory, antiallergic, and antioxidant potentialities rendering it alluring for utilization in food processes [75].

Likewise, plant-mediated biosystems including lettuce, rice, and maize have also been described [78–80]; however, these systems implicate the use of intact transgenic plants, limiting the field cultivation. In a recent study, Han et al. (2020) established a brazzein synthesis platform using carrot cell suspension in a bioreactor. The cell proliferation quickly amplified up to 15 days during the TC12 culture period, reaching the highest cell division rate after 6 days. Compared to control, the gene expression was 2.5-fold and 2.8-fold increased using 220 μM H_2_O_2_ and 50 μM ABA, respectively. The resulting transgenic cells were used in various air-lift bioreactors, where higher biomass (238.9 g L^−1^) was achieved in column bioreactors than balloon and cone bioreactors. The findings reveal the effective production of brazzein in an air-lift bioreactor, which might be useful for the food industry.

## Monellin

Monellin is found in the red berries of the West African plant *Dioscoreophyllum cumminsii* Diels. Purified by Yan et al. [73], monellin is about 3000-times sweeter than sucrose and is utilized in the food industry as a flavor enhancer and sweetener. It exhibits a unique set of advantages over non-natural sweeteners, like safety, low in calories, no introduction of non-natural metabolites in the body, maintenance of amino acid pool balance, and relatively simple cloning into microorganisms [13]. Distinct to single-chain thaumatin, this sugar is composed of two polypeptides of 45 and 50 amino acid residues linked through non-covalent interactions. It loses its sweetening ability over 50°C at acidic pH. To overcome this stability issue, Tyo et al. [52] synthesized single-chain monellin equivalents in which several linkers were used to join two chains. One of these single-chain derivatives was expressed in *E. coli* and found to be a powerful sweetener as well as highly stable under extreme pH and temperature conditions. Attempts have also been devoted to expressing monellin in the *E*. *coli, Candida* utilis and *S. cerevisiae* [81]. Vigues et al. [13] expressed monellin encoding a synthetic gene in an *E. coli* host using a T7 phage promoter. A single-chain monellin gene was constructed to optimize its expression based on the *E. coli* biased codons. The results revealed that monellin production accounts for 45% of the entire soluble proteins. It was subjected to purification yielding 43 mg protein/g dry cell weight.

## Mabinlin

*Capparis masaikai* (a Chinese plant) bears a fruit that comprises a sweet-taste protein [82]. Among the four different sweet-tasting polypeptides in this plant, mabinlin II is regarded as one of the most studied proteins, which consists of two polypeptide chains of 33 and 72 amino acids tightly connected via non-covalent linkages. Compared to sucrose, mabinlin II is about 100-times sweeter on a weight basis. In addition, other variants of mabinlin, namely mabinlins I-1, III, and IV, have also been reported [83]. The discrepancy in the heat-stability profile of all mabinlin variants is attributed to the occurrence of glutamine (heat-unstable homolog) or arginine residue (heat-stable homolog) at position 47 in the β-chain [83]. Researchers have cloned and sequenced the cDNAs indicating all four known mabinlin isoforms (I-1, II, III, and IV) [84].

## Pentadin

*Pentadiplandra brazzeana Baillon* (a climbing shrub) plants present in some tropical African countries (such as Gabon) contain a sweet protein (12 kDa), which was first extracted by Morris and Cagan [105]. Electrophoretic profile studies with and without 2-mercaptoethanol confirmed the presence of subunits joined by disulfide bonds in the mature protein. On a weight basis, the sweetening ability was about 500 times that of sucrose, resembling monellin and higher than thaumatin.

## Curculin

Curculin was initially extracted from the *Curculigo latifolia* plant that cultivates in some regions of Malaysia [108]. It comprises 114 amino acid residues with an estimated molecular weight of 12,491. It is a dimer containing two identical polypeptide chains associated via two disulfide bridges. On a weight basis, curculin is 550-times sweeter than sucrose and is a taste-enhancer with the exceptional capability of turning sour tastes (e.g., lemon) into sweet ones (e.g., orange).

## Miraculin

A taste-modifier protein, namely miraculin was first isolated by Healey [97] in the red berries of a shrub inherent to West Africa (*Richadella dulcifera*). It comprises 191 amino acid residues with an approximated molecular mass of 24,600 [85]. The native form of miraculin is a tetramer connected by several disulfide bonds. Itself, it does not provoke any sweetening response; it can modify a sour taste into a sweet taste like curculin. Boer et al. [57] assembled a synthetic gene encoding miraculin and ligated it into an expression vector of *E. coli*. The cloning results in the synthesis of recombinant miraculin, but a comprehensive characterization was not made [86–91].

## Concluding remarks and outlook

In conclusion, the modern society is becoming more conscious and aware of taking a well-balanced diet for maintaining and promoting their health. Excessive sugar consumption led to several health complications, such as high blood pressure, increased risk of diabetes, and cardiovascular disorders. This challenging situation can effectively be controlled by introducing low-sugar or sugar-free foodstuffs and beverages. Therefore, several food industries have introduced natural sweeteners as alternatives, like sugar alcohols (polyols), high-fructose corn syrup, and most recently, sweet proteins (i.e., thaumatin), which provide consumers with prospective health benefits. Nature is a prolific source of high-value biomolecules, including sweet-tasting proteins, many of which are yet to be explored. Thus, it is highly meaningful to execute in-depth scientific investigation to prove the safety of the natural ingredients as food supplements/additives, such as natural sweeteners.

Expression of the sweet protein in the food safety-grade yeasts like *Y. lipolytica* and *Kluyveromyces marxianus* might represent a meaningful strategy because the latter is thermo-resistance up to 45°C. *Y. lipolytica* has an enormous ability to secrete sweet proteins, which are transported into medium, thus facilitating their purification. The innovative idea is to modify sweet protein by glycosylation via transglycosylase, for example, by the addition of glucose, mannose, galactose, or fucose moiety on the sweet protein to expand their utilization as a potential antibacterial or anti-fungi agent. In addition, coproduction and co-crystalization of sweet protein and functional sugar can lead to functional sugar sweeter than single sugars. For example, erythritol sweetness is 0.7-fold of sucrose and coproducing with sweet proteins; the erythritol sweet potency will be higher than the native form. Currently, *E. coli* and *P. pastoris* are the widely used hosts for expressing sweet protein; however, *E. coli* is considered food unsafe, whereas *P. pastoris* is methanol induced, which is toxic. In contrast, the *Y. lipolytica* and *K. marxianus* are food grade and excellent protein expression hosts. However, extensive work is needed to prove these strains as commercially viable expression hosts [92–113].

## References

[1] Lichtenstein AH, Karpyn A. History and development of the 2015-2020 dietary guidelines for Americans. *Book Chapter*. 2018: 31–46

[2] Joseph JA, Akkermans S, Nimmegeers P, et al. Bioproduction of the recombinant sweet protein thaumatin: current state of the art and perspectives. Front Microbiol. 2019;10:695. 31024485 10.3389/fmicb.2019.00695 PMC6463758

[3] Brisbois TD, Marsden SL, Anderson GH, et al. Estimated intakes and sources of total and added sugars in the Canadian diet. *Nutrients*. 2014;6(5):1899–1912 24815507 10.3390/nu6051899 PMC4042566

[4] You A. Dietary guidelines for Americans. Texas, United States: US Department of Health and Human Services and US Department of Agriculture; 2015.

[5] Agboola DA, Fawibe OO, Ogunyale OG, et al. Botanical and protein sweeteners. *Journal of Advanced Laboratory Research in Biology*. 2014;5(4):169–187.

[6] Kelada KD, Tusé D, Gleba Y, et al. Process Simulation and Techno-Economic Analysis of Large-Scale Bioproduction of Sweet Protein Thaumatin II. Foods. 2021;10(4):838. 33921374 10.3390/foods10040838 PMC8069865

[7] Pearlman M, Obert J, Casey L. The association between artificial sweeteners and obesity. Curr Gastroenterol Rep. 2017;19(12):64. 29159583 10.1007/s11894-017-0602-9

[8] Whitehouse CR, Boullata J, McCauley LA. The potential toxicity of artificial sweeteners. AAOHN J. 2008;56(6):251–261. 18604921 10.3928/08910162-20080601-02

[9] Kokotou MG, Asimakopoulos AG, Thomaidis NS. Artificial sweeteners as emerging pollutants in the environment: analytical methodologies and environmental impact. Anal Methods. 2012;4(10):3057–3070.

[10] Sylvetsky AC, Rother KI. Trends in the consumption of low-calorie sweeteners. Physiol Behav. 2016;164:446–450. 27039282 10.1016/j.physbeh.2016.03.030 PMC5578610

[11] García-Almeida JM, Cornejo-Pareja IM, Muñoz-Garach A, et al. Sweeteners: regulatory Aspects 27. *Sweeteners*. 2018: 613

[12] Nelson G, Chandrashekar J, Hoon MA, et al. An amino-acid taste receptor. Nature. 2002;416(6877):199–202. 11894099 10.1038/nature726

[13] Vigues S, Dotson CD, Munger SD. The receptor basis of sweet taste in mammals. *Chemosensory Systems in Mammals, Fishes, and Insects*. 2008: 20–23

[14] Fernstrom JD, Munger SD, Sclafani A, et al. Mechanisms for sweetness. J Nutr. 2012;142(6):1134S–1141S. 22573784 10.3945/jn.111.149567 PMC3738222

[15] Saraiva A, Carrascosa C, Raheem D, et al. Natural sweeteners: the relevance of food naturalness for consumers, food security aspects, sustainability and health impacts. Int J Environ Res Public Health. 2020;17(17):6285. 10.3390/ijerph17176285 PMC7504156 32872325

[16] Marcus JB. Aging, Nutrition and Taste: nutrition, Food Science and Culinary Perspectives for Aging Tastefully. London, United Kingdom: Academic Press; 2019.

[17] Belloir C, Neiers F, Briand L. Sweeteners and sweetness enhancers. Curr Opin Clin Nutr Metab Care. 2017;20(4):279–285. 28399012 10.1097/MCO.0000000000000377

[18] Laffitte A, Neiers F, Briand L. Characterization of taste compounds: chemical structures and sensory properties. Guichard E, Le Bon AM, Morzel M, et al. Oxford: Wiley-Blackwell; 2016.

[19] World Health Organization. Guideline: sugars intake for adults and children. Geneva, Switzerland: World Health Organization; 2015. 25905159

[20] Grembecka M. Natural sweeteners in a human diet. *Roczniki Państwowego Zakładu Higieny*. 2015;66(3):1516. 26400114

[21] Kroger M, Meister K, Kava R. Low‐calorie sweeteners and other sugar substitutes: a review of the safety issues. Compr Rev Food Sci Food Saf. 2006;5(2):35–47.

[22] Varelis P, Melton L, Shahidi F. Encyclopedia of Food chemistry. Cambridge, Massachusetts, United States: Elsevier; 2018.

[23] Carocho M, Morales P, Ferreira IC. Sweeteners as food additives in the XXI century: a review of what is known, and what is to come. Food Chem Toxicol. 2017;107:302–317. 28689062 10.1016/j.fct.2017.06.046

[24] Shah R, De Jager LS. Recent analytical methods for the analysis of sweeteners in food: a regulatory perspective. *Food safety: innovative analytical tools for food safety assessment*. In: Hoboken, NJ and Scrivener publishing LLC, Beverly. *1st edn* ed. Beverly, Massachusetts, United States: John Wiley & Sons, Inc; 2016. p. 13–32.

[25] Mortensen A. Sweeteners permitted in the European Union: safety aspects. *Scandinavian Journal of Food and Nutrition*. 2006;50(3):104–116

[26] Jiang P, Ji Q, Liu Z, et al. The cysteine-rich region of T1R3 determines responses to intensely sweet proteins. J Biol Chem. 2004;279(43):45068–45075. 15299024 10.1074/jbc.M406779200

[27] Ohta K, Masuda T, Ide N, et al. Critical molecular regions for elicitation of the sweetness of the sweet‐tasting protein, thaumatin I. FEBS J. 2008;275(14):3644–3652. 18544096 10.1111/j.1742-4658.2008.06509.x

[28] van der Wel H, Loeve K. Isolation and characterization of thaumatin I and II, the sweet‐tasting proteins from *Thaumatococcus daniellii* Benth. Eur J Biochem. 1972;31(2):221–225. 4647176 10.1111/j.1432-1033.1972.tb02522.x

[29] Hellfritsch C, Brockhoff A, Stähler F, et al. Human psychometric and taste receptor responses to steviol glycosides. J Agric Food Chem. 2012;60(27):6782–6793. 22616809 10.1021/jf301297n

[30] Pronin AN, Tang H, Connor J, et al. Identification of ligands for two human bitter T2R receptors. Chem Senses. 2004;29(7):583–593. 15337684 10.1093/chemse/bjh064

[31] Kinghorn AD, Kaneda N, Baek NI, et al. Noncariogenic intense natural sweeteners. Med Res Rev. 1998;18(5):347–360. 9735874 10.1002/(sici)1098-1128(199809)18:5<347::aid-med5>3.0.co;2-t

[32] Zemanek EC, Wasserman BP. Issues and advances in the use of transgenic organisms for the production of thaumatin, the intensely sweet protein from Thaumatococcus danielli. Crit Rev Food Sci Nutr. 1995;35(5):455–466. 8573283 10.1080/10408399509527709

[33] Smith J, Hong-Shum L. Food additives data book. Beverly, Massachusetts, United States: John Wiley & Sons; 2011.

[34] Etheridge K. The sales and marketing of talin. (1994). In “Thaumatin”, eds. Witty, M. and Higginbotham, JD. Boca Raton, Florida, United States: CRC Press.

[35] Global Data, 2015, www.globaldata.com/global-data-2015

[36] Recombinant Protein Market Analysis, (2018). Available online at: https://www.coherentmarketinsights.com/market-insight/recombinant-protein-market-1516 [Last accessed 2022 Mar 11].

[37] Demain AL, Vaishnav P. Production of recombinant proteins by microbes and higher organisms. Biotechnol Adv. 2009;27(3):297–306. 19500547 10.1016/j.biotechadv.2009.01.008

[38] Faus I, Pati-o C, Del R’o JL, et al. Expression of a synthetic gene encoding the sweet-tasting protein thaumatin in the filamentous fungus *Penicillium roquefortii*. Biotechnol Lett. 1997;19(12):1185–1191.

[39] Hahm YT, Batt CA. Expression and secretion of thaumatin from Aspergillus oryzae. Agric Biol Chem. 1990;54(10):2513–2520.

[40] Illingworth C, Larson G, Hellekant G. Secretion of the sweet-tasting plant protein thaumatin by *Streptomyces lividans*. J Ind Microbiol. 1989;4(1):37–42.

[41] Weickmann JL. High level expression of thaumatin in *Saccharomyces cerevisiae*. Boca Raton, Florida, United States: CRC Press.*Thaumatin*. 151–169. 1994.

[42] Daniell S, Mellits KH, Faus I, et al. Refolding the sweet-tasting protein thaumatin II from insoluble inclusion bodies synthesised in *Escherichia coli*. Food Chem. 2000;71(1):105–110.

[43] Hung CY, Cheng LH, Yeh CM. Functional expression of recombinant sweet-tasting protein brazzein by *Escherichia coli* and *Bacillus licheniformis*. Food Biotechnol. 2019;33(3):251–271.

[44] Terpe K. Overview of bacterial expression systems for heterologous protein production: from molecular and biochemical fundamentals to commercial systems. Appl Microbiol Biotechnol. 2006;72(2):211–222. 16791589 10.1007/s00253-006-0465-8

[45] Swartz JR. *Escherichia coli* recombinant DNA technology. *Escherichia coli and Salmonella: cellular and molecular biology*. 1996;2:1693–1711.

[46] Jenkins N, Curling EM. Glycosylation of recombinant proteins: problems and prospects. Enzyme Microb Technol. 1994;16(5):354–364. 7764790 10.1016/0141-0229(94)90149-x

[47] Chen R. Bacterial expression systems for recombinant protein production: e. coli and beyond. Biotechnol Adv. 2012;30(5):1102–1107. 21968145 10.1016/j.biotechadv.2011.09.013

[48] Yeh CM, Kao BY, Peng HJ. Production of a recombinant type 1 antifreeze protein analogue by *L. lactis* and its applications on frozen meat and frozen dough. J Agric Food Chem. 2009;57(14):6216–6223. 19545118 10.1021/jf900924f

[49] Faus I, Patiño C, Del R’o JL, et al. Expression of a Synthetic Gene Encoding the Sweet-Tasting Protein Thaumatin in *Escherichia coli*. Biochem Biophys Res Commun. 1996;229(1):121–127. 8954093 10.1006/bbrc.1996.1767

[50] Goffeau A, Barrell BG, Bussey H, et al. Life with 6000 genes. Science. 1996;274(5287):546–567. 8849441 10.1126/science.274.5287.546

[51] Tang H, Bao X, Shen Y, et al. Engineering protein folding and translocation improves heterologous protein secretion in *Saccharomyces cerevisiae*. Biotechnol Bioeng. 2015;112(9):1872–1882. 25850421 10.1002/bit.25596

[52] Tyo KEJ, Liu Z, Magnusson Y, et al. Impact of protein uptake and degradation on recombinant protein secretion in yeast. Appl Microbiol Biotechnol. 2014;98(16):7149–7159. 24816620 10.1007/s00253-014-5783-7

[53] Mattanovich D, Sauer M, Gasser B. Yeast biotechnology: teaching the old dog new tricks. Microb Cell Fact. 2014;13(1):1–5. 24602262 10.1186/1475-2859-13-34 PMC3975642

[54] Edens L, Van der Wel H. Microbial synthesis of the sweet-tasting plant protein thaumatin. Trends Biotechnol. 1985;3(3):61–64.

[55] Lee H-M, Park S-W, Lee S-J, et al. Optimized production and quantification of the tryptophan-deficient sweet-tasting protein brazzein in *Kluyveromyces lactis*. *Preparative Biochemistry and Biotechnology*. 2019;49(8):790–799 31140364 10.1080/10826068.2019.1621892

[56] Park SW, Kang BH, Lee HM, et al. Efficient brazzein production in yeast (*Kluyveromyces lactis*) using a chemically defined medium. Bioprocess Biosyst Eng. 2021;44(4):913–925. 33502625 10.1007/s00449-020-02499-y

[57] Böer E, Gellissen G, Kunze G. Arxula adeninivorans. *Production of recombinant proteins*. 2005: 89–110

[58] Gellissen G, Ed. Production of recombinant proteins: novel microbial and eukaryotic expression systems. Beverly, Massachusetts, United States: John Wiley & Sons; 2006.

[59] Kang HA, Gellissen G. Hansenula polymorpha. *Production of Recombinant Proteins*. 2005: 111–142

[60] Madzak C, Nicaud JM, Gaillardin C, et al. Production of recombinant proteins: novel microbial and eukaryotic expression systems. Wiley-VCH: Weinheim. 2005. 163–189. 10.1002/3527603670.ch8

[61] Cereghino GPL, Cereghino JL, Ilgen C, et al. Production of recombinant proteins in fermenter cultures of the yeast *Pichia pastoris*. Curr Opin Biotechnol. 2002;13(4):329–332. 12323354 10.1016/s0958-1669(02)00330-0

[62] Hellwig S, Emde F, Raven NP, et al. Analysis of single‐chain antibody production in *Pichia pastoris* using on‐line methanol control in fed‐batch and mixed‐feed fermentations. Biotechnol Bioeng. 2001;74(4):344–352. 11410859

[63] Macauley‐Patrick S, Fazenda ML, McNeil B, et al. Heterologous protein production using the *Pichia pastoris* expression system. *Yeast*. 2005;22(4):249–270 15704221 10.1002/yea.1208

[64] Cereghino JL, Cregg JM. Heterologous protein expression in the methylotrophic yeast *Pichia pastoris*. FEMS Microbiol Rev. 2000;24(1):45–66. 10640598 10.1111/j.1574-6976.2000.tb00532.x

[65] Inan M, Aryasomayajula D, Sinha J, et al. Enhancement of protein secretion in *Pichia pastoris* by overexpression of protein disulfide isomerase. Biotechnol Bioeng. 2006;93(4):771–778. 16255058 10.1002/bit.20762

[66] Robinson AS, Hines V, Wittrup KD. Protein disulfide isomerase overexpression increases secretion of foreign proteins in *Saccharomyces cerevisiae*. *Bio technology*. 1994;12(4):381–384 10.1038/nbt0494-381 7764684

[67] Caldwell JE, Abildgaard F, Džakula Ž, et al. Solution structure of the thermostable sweet-tasting protein brazzein. Nat Struct Biol. 1998;5(6):427–431. 9628478 10.1038/nsb0698-427

[68] Assadi-Porter FM, Aceti DJ, Cheng H, et al. Efficient production of recombinant brazzein, a small, heat-stable, sweet-tasting protein of plant origin. Arch Biochem Biophys. 2000;376(2):252–258. 10775410 10.1006/abbi.2000.1725

[69] Ming D, Hellekant G. Brazzein, a new high-potency thermostable sweet protein from *Pentadiplandra brazzeana* B. FEBS Lett. 1994;355(1):106–108. 7957951 10.1016/0014-5793(94)01184-2

[70] Tomes DT. Methods and compositions for production of plant foodstuffs with enhanced sweet component flavor. *PCT Patent WO*. 1997;97:42333.

[71] Hellekant BG, Ming D (1994). Brazzein sweetener. *US Patent*, 5346998.

[72] Berlec A, Jevnikar Z, Majhenič AČ, et al. Expression of the sweet-tasting plant protein brazzein in *Escherichia coli and* Lactococcus lactis: a path toward sweet lactic acid bacteria. Appl Microbiol Biotechnol. 2006;73(1):158–165. 16703320 10.1007/s00253-006-0438-y

[73] Yan S, Song H, Pang D, et al. Expression of plant sweet protein brazzein in the milk of transgenic mice. *Plos one*. 2013;8(10):e76769 24155905 10.1371/journal.pone.0076769 PMC3796561

[74] Yun CR, Kong JN, Chung JH, et al. Improved secretory production of the sweet-tasting protein, brazzein, in *Kluyveromyces lactis*. J Agric Food Chem. 2016;64(32):6312–6316. 27465609 10.1021/acs.jafc.6b02446

[75] Chung JH, Kong JN, Choi HE, et al. Antioxidant, anti-inflammatory, and anti-allergic activities of the sweet-tasting protein brazzein. Food Chem. 2018;267:163–169. 29934152 10.1016/j.foodchem.2017.06.084

[76] Jo HJ, Noh JS, Kong KH. Efficient secretory expression of the sweet-tasting protein brazzein in the yeast *Kluyveromyces lactis*. Protein Expr Purif. 2013;90(2):84–89. 23684772 10.1016/j.pep.2013.05.001

[77] Lee J-W, Cha J-E, Jo H-J, et al. Multiple mutations of the critical amino acid residues for the sweetness of the sweet-tasting protein, brazzein. Food Chem. 2013;138(2–3):1370–1373. 23411256 10.1016/j.foodchem.2012.10.140

[78] Jung YJ, Kang KK. Stable expression and characterization of brazzein, thaumatin and miraculin genes related to sweet protein in transgenic lettuce. *Journal of Plant Biotechnology*. 2018;45(3):257–265

[79] Lamphear BJ, Barker DK, Brooks CA, et al. Expression of the sweet protein brazzein in maize for production of a new commercial sweetener. Plant Biotechnol J. 2004;3(1):103–114. 10.1111/j.1467-7652.2004.00105.x 17168903

[80] Lee YR, Akter S, Lee IH, et al. Stable expression of brazzein protein, a new type of alternative sweetener in transgenic rice. *Journal of Plant Biotechnology*. 2018;45(1):63–70

[81] Kondo K, Miura Y, Sone H, et al. High-level expression of a sweet protein, monellin, in the food yeast *Candida utilis*. Nat Biotechnol. 1997;15(5):453–457. 9131625 10.1038/nbt0597-453

[82] Liu X, Maeda S, Hu Z, et al. Purification, complete amino acid sequence and structural characterization of the heat‐stable sweet protein, mabinlin II. Eur J Biochem. 1993;211(1‐2):281–287. 8425538 10.1111/j.1432-1033.1993.tb19896.x

[83] Nirasawa S, Nishino T, Katahira M, et al. Structures of heat‐stable and unstable homologues of the sweet protein mabinlin. The difference in the heat stability is due to replacement of a single amino acid residue. Eur J Biochem. 1994;223(3):989–995. 8055976 10.1111/j.1432-1033.1994.tb19077.x

[84] Sun S, Xiong L, Hu Z, et al. (1997) Recombinant sweet protein mabinlin. PCT Patent WO 97/00945

[85] Theerasilp S, Hitotsuya H, Nakajo S, et al. Complete amino acid sequence and structure characterization of the taste-modifying protein, miraculin. J Biol Chem. 1989;264(12):6655–6659. 2708331

[86] Abad S, Nahalka J, Winkler M, et al. High-level expression of Rhodotorula gracilis d-amino acid oxidase in *Pichia pastoris*. Biotechnol Lett. 2011;33(3):557–563. 21053050 10.1007/s10529-010-0456-9

[87] EFSA Panel on Additives and Products or Substances used in Animal Feed (FEEDAP). Scientific Opinion on the Safety and Efficacy of thaumatin for all animal species. EFSA J. 2011;9(9):2354.

[88] Gellissen G, Kunze G, Gaillardin C, et al. New yeast expression platforms based on methylotrophic *Hansenula polymorpha* and *Pichia pastoris* and on dimorphic *Arxula adeninivorans* and *Yarrowia lipolytica*–a comparison. FEMS Yeast Res. 2005a;5(11):1079–1096. 16144775 10.1016/j.femsyr.2005.06.004

[89] Gellissen G, Strasser AW, Suckow M. Key and criteria to the selection of an expression platform. *Production of recombinant proteins–Novel microbial and eukaryotic expression systems*. 2005b: 1–5

[90] Masuda T. Sweet-tasting protein thaumatin: physical and chemical properties. In: Merillon J-M, Ramawat KG, editors. Sweeteners: Pharmacology, Biotechnology, and Applications, Springer, Cham. 2018. p. 493–523.

[91] Ohta K, Masuda T, Tani F, et al. The cysteine-rich domain of human T1R3 is necessary for the interaction between human T1R2–T1R3 sweet receptors and a sweet-tasting protein, thaumatin. Biochem Biophys Res Commun. 2011b;406(3):435–438. 21329673 10.1016/j.bbrc.2011.02.063

[92] Chen Z, Cai H, Lu F, et al. High-level expression of a synthetic gene encoding a sweet protein, monellin, in *Escherichia coli*. Biotechnol Lett. 2005;27(22):1745–1749. 16314964 10.1007/s10529-005-3544-5

[93] Edens L, Heslinga L, Klok R, et al. Cloning of cDNA encoding the sweet-tasting plant protein thaumatin and its expression in *Escherichia coli*. *Gene*. 1982;18(1):1–12 7049841 10.1016/0378-1119(82)90050-6

[94] Faus I. Recent developments in the characterization and biotechnological production of sweet-tasting proteins. Appl Microbiol Biotechnol. 2000;53(2):145–151. 10709975 10.1007/s002530050001

[95] Faus I, Del Moral C, Adroer N, et al. Secretion of the sweet-tasting protein thaumatin by recombinant strains of Aspergillus Niger var. awamori. Appl Microbiol Biotechnol. 1998;49(4):393–398. 9615480 10.1007/s002530051188

[96] Harada S, Otani H, Maeda S, et al. Crystallization and preliminary X-ray diffraction studies of curculin: a new type of sweet protein having taste-modifying action. J Mol Biol. 1994;238(2):286–287. 8158656 10.1006/jmbi.1994.1289

[97] Healey RD, Lebhar H, Hornung S, et al. An improved process for the production of highly purified recombinant thaumatin tagged-variants. Food Chem. 2017;237:825–832. 28764073 10.1016/j.foodchem.2017.06.018

[98] Higginbotham JD, Snodin DJ, Eaton KK, et al. Safety evaluation of thaumatin (Talin protein). Food Chem Toxicol. 1983;21(6):815–823. 6686588 10.1016/0278-6915(83)90218-1

[99] Inglett GE, MAY JF. Serendipity berries–source of a new intense sweetener. J Food Sci. 1969;34(5):408–411.

[100] Jain T, Grover K. Sweeteners in human nutrition. Int J Health Sci Res. 2015;5(5):439–451.

[101] Kim SH, Kang CH, Kim R, et al. Redesigning a sweet protein: increased stability and renaturability. Protein Eng Des Sel. 1989;2(8):571–575. 10.1093/protein/2.8.571 2813335

[102] Kurihara Y. Sweet proteins in general. Thaumatin. 1994;1–18.

[103] Maehashi K, Udaka S. Sweetness of lysozymes. Biosci Biotechnol Biochem. 1998;62(3):605–606. 9571795 10.1271/bbb.62.605

[104] Masuda T, Tamaki S, Kaneko R, et al. Cloning, expression and characterization of recombinant sweet‐protein thaumatin II using the methylotrophic yeast *Pichia pastoris*. Biotechnol Bioeng. 2004;85(7):761–769. 14991654 10.1002/bit.10786

[105] Morris JA, Cagan RH. Purification of monellin, the sweet principle of *Dioscoreophyllum cumminsii*. Biochim Biophys Acta. 1972;261(1):114–122. 5012458 10.1016/0304-4165(72)90320-0

[106] Nabors L, Gelardi R. Alternative sweeteners: an overview. *Alternative Sweeteners*. 2001;2:1–10.

[107] Neiers F, Belloir C, Poirier N, et al. Comparison of different signal peptides for the efficient secretion of the sweet-tasting plant protein brazzein in *Pichia pastoris*. Life. 2021;11(1):46. 33450886 10.3390/life11010046 PMC7828362

[108] Ohta K, Masuda T, Tani F, et al. Introduction of a negative charge at Arg82 in thaumatin abolished responses to human T1R2–T1R3 sweet receptors. Biochem Biophys Res Commun. 2011a;413(1):41–45. 21867681 10.1016/j.bbrc.2011.08.033

[109] Overbeeke N. Synthesis and processing of thaumatin in yeast. Biotechnology. 1989;13:305–318. 2679930

[110] Takahashi N, Hitotsuya H, Hanzawa H, et al. Structural study of asparagine-linked oligosaccharide moiety of taste-modifying protein, miraculin. J Biol Chem. 1990;265(14):7793–7798. 2335505

[111] Theerasilp S, Kurihara Y. Complete purification and characterization of the taste-modifying protein, miraculin, from miracle fruit. J Biol Chem. 1988;263(23):11536–11539. 3403544

[112] Wel HVD, Larson G, Hladik A, et al. Isolation and characterization of pentadin, the sweet principle of *Pentadiplandra brazzeana* Baillon. Chem Senses. 1989;14(1):75–79.

[113] Yamashita H, Theerasilp S, Aiuchi T, et al. Purification and complete amino acid sequence of a new type of sweet protein taste-modifying activity, curculin. J Biol Chem. 1990;265(26):15770–15775. 2394746

